# Influence of IGF-I serum concentration on muscular regeneration capacity in patients with sarcopenia

**DOI:** 10.1186/s12891-021-04699-3

**Published:** 2021-09-20

**Authors:** Stefanie Jarmusch, Lisa Baber, Martin Bidlingmaier, Uta Ferrari, Fabian Hofmeister, Stefan Hintze, Stefan Mehaffey, Peter Meinke, Carl Neuerburg, Benedikt Schoser, Fabiana Tanganelli, Michael Drey

**Affiliations:** 1 Department of Medicine IV, Geriatrics, University Hospital of LMU Munich, Munich, Germany; 2 Department of Medicine IV, Endocrinological Laboratory, University Hospital of LMU Munich, Munich, Germany; 3 Friedrich-Baur-Institute, Department of Neurology, University Hospital of LMU Munich, Munich, Germany; 4 Department of Orthopaedics and Trauma Surgery, Musculoskeletal University Center Munich (MUM), University Hospital of LMU Munich, Munich, Germany

**Keywords:** Insulin-like growth factor 1, MUNIX, Muscle atrophy, Denervation

## Abstract

**Background:**

Previous research has described a neuroprotective effect of IGF-I, supporting neuronal survival, axon growth and proliferation of muscle cells. Therefore, the association between IGF-I concentration, muscle histology and electrophysiological markers in a cohort of patients with sarcopenia dares investigation.

**Methods:**

Measurement of serum concentrations of IGF-I and binding partners, electromyographic measurements with the MUNIX (Motor Unit Number Index) method and muscle biopsies were performed in 31 patients with acute hip fracture older age 60 years. Molecular markers for denervation (neural cell adhesion molecule NCAM) and proliferation markers (Ki67) were assessed by immunofluorescence staining of muscle biopsy tissue. Skeletal muscle mass by bioelectrical impedance analysis and hand-grip strength were measured to assess sarcopenia status according to EWGSOP2 criteria.

**Results:**

Thirty-one patients (20 women) with a mean age of 80.6 ± 7.4 years were included. Concentrations of IGF-I and its binding partners were significantly associated with sarcopenia (ß = − 0.360; *p* = 0.047) and MUNIX (ß = 0.512; *p* = 0.005). Further, expression of NCAM (ß = 0.380; *p* = 0.039) and Ki67 (ß = 0.424; *p* = 0.022) showed significant associations to IGF-I concentrations.

**Conclusions:**

The findings suggest a pathogenetic role of IGF-I in sarcopenia based on muscle denervation.

**Supplementary Information:**

The online version contains supplementary material available at 10.1186/s12891-021-04699-3.

## Background

Sarcopenia, the progressive and age-associated loss of skeletal muscle mass and function [1] is a major reason for dependency and mortality in ageing societies. It is affecting around 6 to 22% of those older than 65 years [2]. Consequential falls, fractures, immobility and mortality add up to at least US $12–26 billion in health care costs annually in the United States of America alone [3] and thus place an enormous economic burden on the healthcare systems. As populations globally continue to age, wide-ranging research has been conducted in this field, yet the pathogenesis of sarcopenia is still not fully understood. It is believed to be a multifactorial pathogenesis, including several different factors such as an imbalance in protein metabolism and catabolism [4], satellite cell dysfunction and following type II muscle fiber atrophy [5], but also physical inactivity [6]. Dysregulation or reduction of testosterone, estrogen, growth hormone (GH) and insulin-like growth factor 1 (IGF-I) [7], as well as neurodegenerative factors, such as the loss of motor neurons and denervation resulting in muscle atrophy play another crucial role [8]. Beyond motoneuron degeneration or peripheral nerve affection, denervation of muscle fibers can result from direct damage, atrophy or disruption of the neuromuscular junction and leads to muscle atrophy and possible myofiber death [9]. Several different electrophysiological techniques to estimate motor unit numbers have identified an association between low motor unit numbers and sarcopenia [10–12]. Histological evaluation of muscle tissue usually includes measurement of expression of the usually used histological marker for denervated myofibers neural cell adhesion molecule (NCAM). It is unclear whether the reinnervation capacity is reduced in patients with sarcopenia and what specific role IGF-I plays in that regard. IGF-I is known to be an important anabolic hormone in muscle metabolism and an important modulator of muscle growth and regeneration. IGF-I is produced in the liver in response to GH, a hormone produced by the pituitary gland. This hormonal regulation is referred to as the GH/IGF-I axis. Circulating IGF-I is usually bound in a ternary complex consisting of the acid labile subunit (ALS) and an insulin-like growth factor binding protein (IGFBP), whereof six different IGFBPs (IGFBP-1 to IGFBP-6) are existing with different tissue expression patterns [13]. IGF-I production can also be detected in the skeletal muscle itself [14] as a response to GH, testosterone or muscle stretch [15]. It promotes hypertrophic effects in skeletal muscle via proliferation of satellite cells, stimulation of protein synthesis [15] and myoblast proliferation and differentiation [16]. The antigen Ki67 is a nuclear protein that is associated with cellular proliferation and a suitable molecule to study proliferation in muscle cells [17]. Transgenic mice overexpressing a local muscle isoform of IGF-I display massive muscle hypertrophy, supporting the anabolic effect of IGF-I [18]. GH secretion and subsequent IGF-I production is markedly reduced while aging [15] with consequently poor muscle strength and poor physical performance [19, 20]. Another important function of IGF-I is its growth promoting effect on the peripheral and central nervous system. As a growth factor, it has potent effects on neurons, including enhancing neuronal survival, neurite formation and outgrowth in motoneurons [21, 22]. In particular, IGF-I has a stimulating effect on axonal sprouting and damage repair [22, 23]. It therefore has been considered and tested as a potential therapeutic drug in neurodegenerative diseases like amyotrophic lateral sclerosis [24–26].

Since IGF-I is a key growth factor in muscle tissue and also in the peripheral and central nervous system by promoting neuronal survival, this study focuses on the association of IGF-I concentrations and number of motor units measured by MUNIX, and expression of NCAM and Ki67 in patients with sarcopenia.

## Methods

### Participants

In collaboration with the Department of General-, Trauma- and Reconstructive Surgery of the University Hospital Munich, we recruited 31 hip fracture patients undergoing surgery aged 60 years or above from November 2017 to March 2019. Exclusion criteria included age younger than 60 years, specific neuromuscular diseases (e.g. myasthenia gravis, muscular dystrophy, amyotrophic lateral sclerosis, polio, myositis), dementia, chronic inflammatory diseases (e.g. Crohn’s disease, ulcerative colitis, rheumatoid arthritis), systemic corticosteroid therapy, and cancer therapy in the last 5 years. The study protocol was approved by the Ethics Committee of LMU Munich (IRB-No. 328–15). All participants gave their written informed consent before surgery.

### Sarcopenia assessment

Sarcopenia was defined according to the European Working Group on Sarcopenia in Older People 2 (EWGSOP2) criteria [27]. Bioelectrical impedance analysis (BIA) was done under standard conditions, with the patient in a supine position and surface electrodes placed on the wrist and ankle contralateral to the side of the fracture. Appendicular lean mass (aLM) was estimated using the equation developed by Sergi et al. [28]. The skeletal muscle index (SMI, [kg/m^2^]) was calculated by dividing aLM by squared body height. Thresholds for low muscle mass were defined as SMI below 7 kg/m^2^ in men and 5.5 kg/m^2^ in women. Handgrip strength was assessed with a Saehan DHD-1 Digital Hand Dynamometer, with the patient sitting upright and the arm held in a 90-degree flexion. Three consecutive measurements of both hands were taken and the maximum value was obtained. Thresholds for handgrip strength were 27 kg for men and 16 kg for women [27]. Patients were classified as being sarcopenic by low muscle muss and low handgrip strength. A z-score combining handgrip strength and muscle mass was calculated separately for men [z-score sarcopenia_men_ = [(27 – handgrip strength)/SD (hand-grip strength)] + [(7.0 – SMI)/SD (SMI)] and women [z-score sarcopenia_women_ = [(16 – hand-grip strength)/SD (hand-grip strength)] + [(5.5 – SMI)/SD (SMI)]. The higher the Z-score, the more sarcopenic the patient. Measurements were carried out between two and 7 days after surgery (mean value: 4 days).

### Muscle biopsies

Open biopsy of the vastus lateralis muscle was performed during surgery for hip fracture. The biopsy was directly cryo-conserved. Muscle tissue blocks were cut to 10 μm tissue sections on a cryostat (HM505E; Micron, Walldorf, Germany) at − 26° Celsius and mounted on glass slides (double frosted microscope slides; Fisher Scientific), air dried for 2 h and stored at − 80° Celsius.

### Immunofluorescence staining for NCAM and Ki67

Muscle sections were cautiously defrosted from − 80° Celsius and allowed to air dry for 15 min at room temperature. For NCAM staining, the sections underwent several washing cycles with blocking solution (TBS_t 0,1%_ and Gelatin Fish 0,9%) and were incubated with the primary antibody for NCAM overnight (polyclonal rabbit anti-NACM (ab5032, 1:100; Millipore Sigma). After incubation and washing cycles with TBS _0,1%_, the tissue sections were incubated with the second antibody (anti-rabbit Alexa Fluor plus 594, 1:500; Invitrogen) and afterwards covered with fluorescence mounting medium with DAPI (Vertor Laboratories, Inc. Burlingame, CA 94010). NCAM expression was detected by counting randomly selected 100 to 150 cells with a Zeiss Fluorescence microscope (Zeiss Axiovert 200 M fluorescence microscope, Zeiss, Oberkochen, Germany).

Assessment of proliferation of the muscle cells was determined using an immunohistochemistry assay with the proliferation marker Ki67 on myoblast cell culture cells gained from muscle biopsy tissue. Cells were washes 3 times with 1x Tris-buffered Saline (TBS) and incubated in blocking solution, containing TBS/Tween (20%) and Fish Gelantine (1%) in a ratio of 10:1 followed by overnight incubation in the primary antibody solution, containing Ki67 (1:100 Donkey anti Rabbit IgG Alexa Fluor™ Plus 594, Invitrogen) dissolved in blocking solution. After 3 washing cycles, the cells incubated for 1 h in the secondary antibody solution (1:500 Donkey anti-Mouse IgG [H + L] Highly Cross-Adsorbed Secondary Antibody, Alexa Fluor Plus 594), dissolved in blocking solution. Coverslips were then covered with 30 μl Mounting Medium containing DAPI (Vectashield) and incubated in a darkened staining chamber. Immunofluorescence images were acquired using a microscope (Olympus fluoview FV1000) and an oil immersion 20x objective and the Olympus Fluoview Software (version 4.2.a). The optical fields were selected randomly to count 100 to 150 cells with Image J (Version 2.0.0).

### MUNIX/MUSIX measurements

The MUNIX technique is an electromyographic method for estimation of number and size of motor units (MUNIX = Motor unit number index, MUSIX = Motor unit size index) based on a mathematical model first described by Nandedkar [29]. Electrodes (15 mm × 20 mm; CareFusion, Middleton, Wisconsin, USA) were placed over the motor point of the hypothenar muscle (active electrode), on the distal hypothenar phalanx (reference electrode) and on the wrist (ground electrode) of the non-fractured patient side. An EMG device (NATUS, Pleasaton, CA, USA) was used. Briefly, the MUNIX procedure consists of 3 steps. The first step is the measurement of the compound muscle action potential (CMAP) by supramaximal stimulation of the ulnar nerve. Three consecutive supramaximal stimulations were performed to obtain the highest CMAP amplitude used for calculation of its amplitude, area and power. Secondly, the surface electromyographic interference patterns (SIPs) are measured. Patients were instructed to exert and maintain an isometric contraction of the hypothenar muscle at varying levels and repeated several times in order to obtain 18 different SIPs. CMAP and SIP were recorded using a bandpass filter setting of 3–3000 Hz. Area and power of the signals were calculated for calculation of MUNIX (MUNIX = A ● (20*mVms*)^α^). MUSIX is then calculated by dividing the CMAP amplitude by MUNIX: *MUSIX*= *Amplitude* (*CMAP*)/*MUNIX* [μV]. It should be noticed that MUNIX and MUSIX are no absolute values but equivalents of number and size of a motor unit. To prevent biases caused by artifacts in the SIP epochs, only those SIPs eligible for the following criteria, as proposed by Nandedkar [30], were accepted for analysis: (a) SIP area > 20mVms; (b) Ideal case motor unit count (ICMUC) < 100; (c) SIP area/CMAP area > 1. MUNIX values < 80 and MUSIX values > 100 μV were classified as low or pathological [30]. Further background information on the mathematical model can be found in earlier publications by Drey et al. [12] and in the Additional file 1.

### Laboratory measurements

Blood samples for measurement of serum concentrations of IGF-I, IGFBP-3 and ALS as parameter for the somatotropic axis were obtained on the third postoperative day. After centrifugation, the serum was stored at − 80° Celsius until analysis. Serum hormone concentrations (ng/ml) of IGF-I and IGFBP-3 were measured at the Endocrine Laboratory of the University Hospital Munich (KUM, Germany) using the IDS-iSYS automated chemiluminescent assay system (Immunodiagnostic System Ltd., Boldon, England, UK). Validation data for all assays and reference intervals have been published elsewhere [31, 32]. The assays are calibrated against the latest recombinant standards (98/574 for GH and 02/254 for IGF-I). Serum ALS concentrations were measured in duplicate by a sandwich-type immunofluorometric assay using monoclonal antibodies directed against specific N- and C-terminal oligopeptides [33]. A serum pool of healthy male volunteers was used for calibration and assigned 1000 U/L. The assay range is 100–1500 U/L, and intra- and inter-assay CVs are below 9%.

### Statistical analysis

**Fig. 1:**
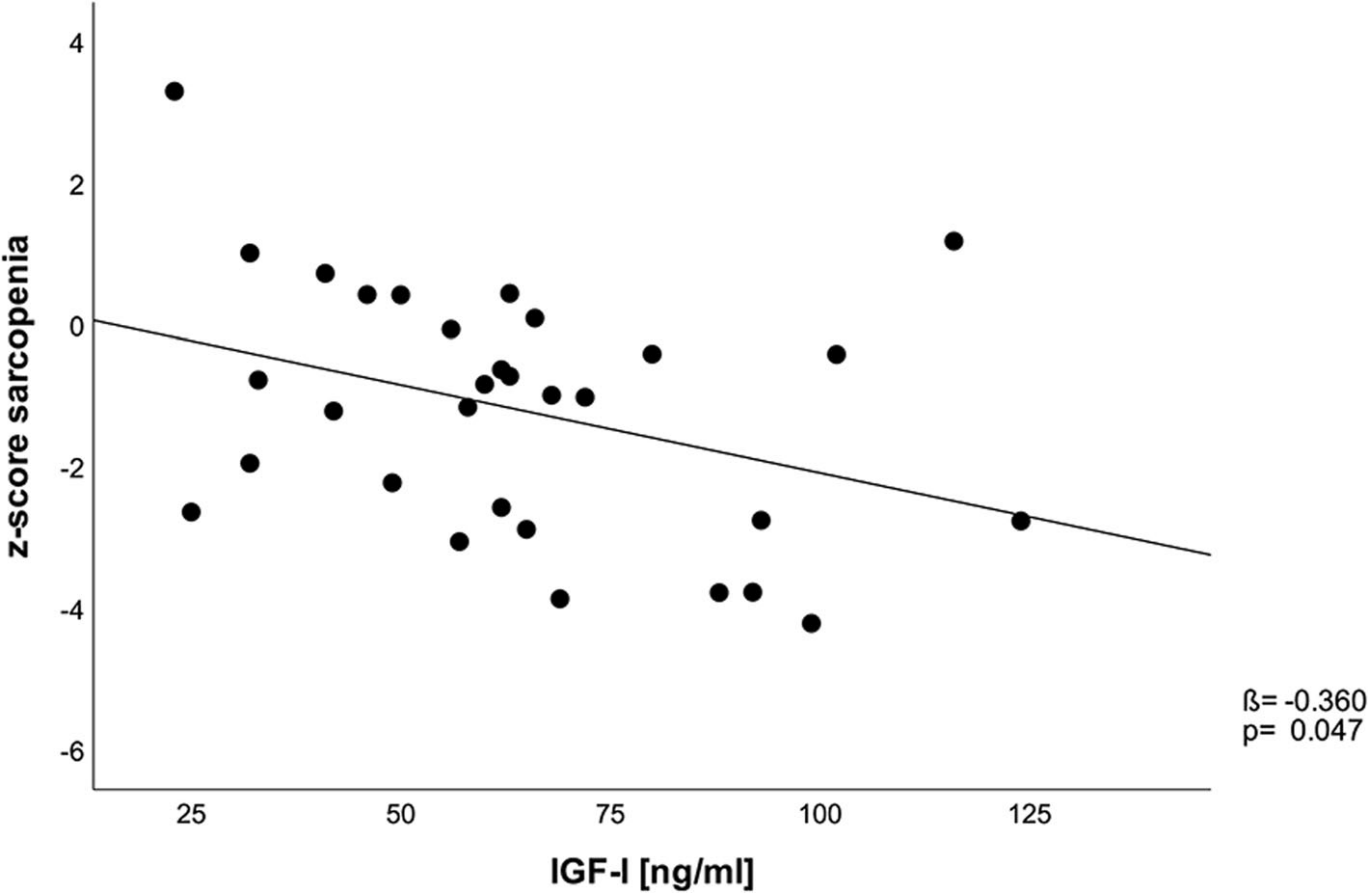
Relation of IGF-I serum concentrations and sarcopenia severity, represented by z-score sarcopenia. Legend: IGF-I = insulin-like growth factor 1

**Fig. 2:**
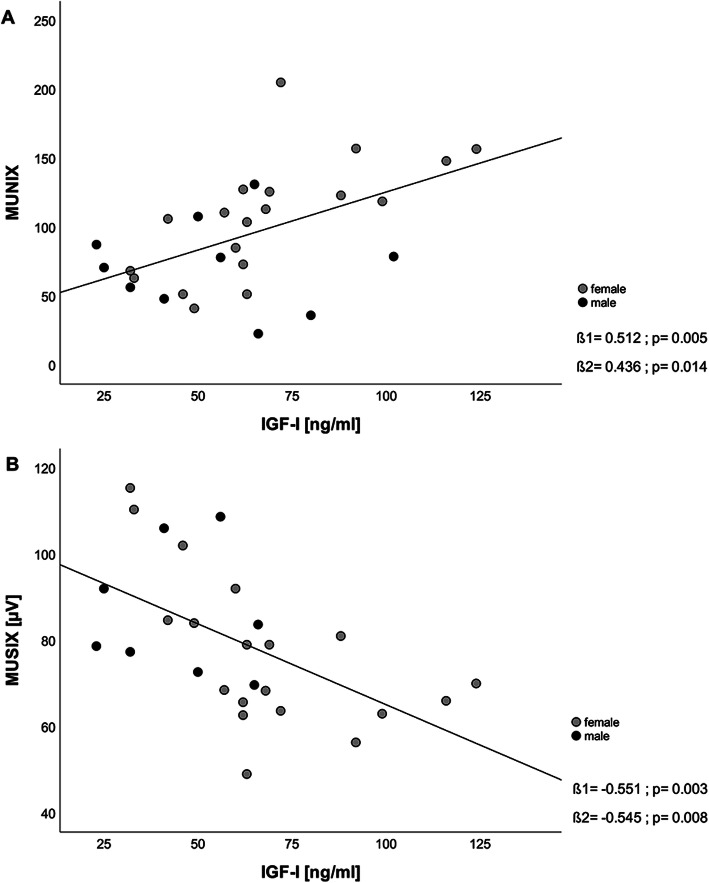
Relation of IGF-I serum concentrations and MUNIX (**A**) and MUSIX values (**B**) stratified by sex. Legend: Two participants did not tolerate the MUNIX procedure and two MUSIX results had to be excluded from analysis due to unreliable measurement. ß1 = unadjusted regression coefficient, ß2 = regression coefficient adjusted for sex, IGF-I = insulin-like growth factor 1, MUNIX = Motor unit number index, MUSIX = Motor unit size index

**Fig. 3:**
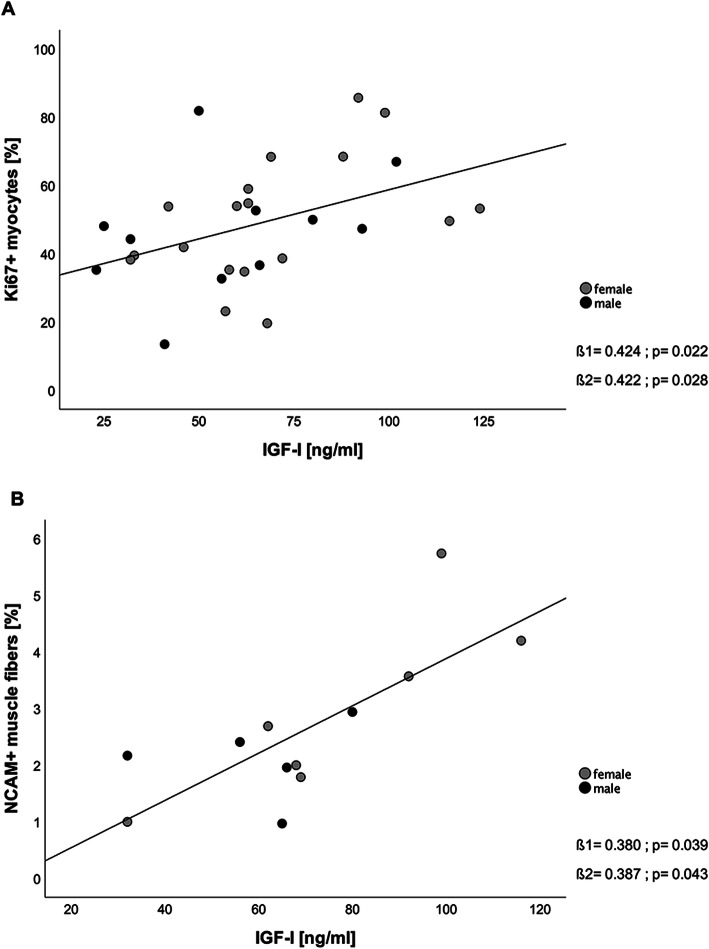
Relation of IGF-I serum concentrations and percentage of Ki67+ expressing myocytes (**A**) and NCAM+ positive muscle fibers (**B**). Legend: Two missing values for Ki67 staining and one missing for NCAM expression due to insufficient antibody binding. Only in 12 of 31 patients NCAM expression was found, as displayed here. ß1 = unadjusted regression coefficient, ß2 = regression coefficient adjusted for sex, IGF-I = insulin-like growth factor 1, NCAM = neural cell adhesion molecule

Statistical analyses were performed using IBM software SPSS v26.0 (IBM-SPSS Inc., Chicago, II, USA). Participants’ characteristics were expressed as mean values and their standard deviation (Table 1). Group differences were calculated by Student’s t-test. Scatter plots and Pearson’s correlation coefficients were shown to visualize correlations between IGF-I and Z-score sarcopenia, MUNIX, MUSIX, NCAM and Ki67 (Figs. 1, 2 and 3). As our study cohort comprise 20 women and only 11 men, multiple linear regression analysis was used to adjust the relations between IGF-I and MUNIX, MUSIX, NCAM and Ki67 for gender (Figs. 2 and 3). In all these models, IGF-I is the independent variable, whereas MUNIX, MUSIX, NCAM and Ki67 are dependent variables. The adjustment is not necessary for the relation between IGF-I and Z-score sarcopenia, as gender is reflected in the Z-score (Fig. 1). Statistical significance was set at *p* < 0.05 for all analyses.

**Table 1:** Patients characteristics stratified for gender

	Total sample *n* = 31	Female *n* = 20	Male *n* = 11	*p*-Value
Age [years]	80.6 (7.4)	79.6 (8.4)	82.5 (5.2)	0.306
Handgrip strength [kg]	23.2 (8.9)	20.6 (6.9)	27.8 (10.6)	**0.030**
SMI [kg/m^2^]	6.9 (1.2)	6.7 (1.2)	7.3 (1.3)	0.163
z-score sarcopenia	−1.2 (1.8)	−1.7 (1.6)	−0.3 (1.9)	**0.040**
IGF-I [ng/ml]	64.1 (25.6)	67.8 (25.0)	57.5 (26.7)	0.296
IGFBP-3 [ng/ml]	1577 (544)	1729 (542)	1300 (446)	**0.033**
ALS [mU/ml]	259 (131)	301 (126)	182 (104)	**0.013**
MUNIX	95 (43)	107 (43)	72 (33)	**0.032**
MUSIX [μV]	87 (35)	77 (18)	106 (51)	**0.030**
Ki67+ myocytes [%]	48.6 (17.9)	50.0 (18.2)	46.3 (18.0)	0.600
NCAM+ fibers [%]	1.1 (1.6)	1.1 (1.8)	1.0 (1.2)	0.797

Mean (SD) unless stated otherwise. Two patients did not tolerate the MUNIX procedure. Two missing measurements for Ki67 staining and 1 missing for NCAM expression due to insufficient antibody binding. *SMI* Skeletal muscle index, *IGF-I* Insulin-like growth factor 1, *IGFBP-3* Insulin-like growth factor binding protein 3, *ALS* Acid labile subunit, *MUNIX* Motor unit number index, *MUSIX* Motor unit size index, *NCAM* neural cell adhesion molecule. *p*-values between groups were calculated using two sample-t-test. *p*-Values <.05 are bold

## Results

The participants’ characteristics are shown in Table 1. A total of 31 patients (20 women) with a mean age of 80.6 ± 7.4 years were recruited. Two participants did not tolerate the MUNIX procedure. Due to insufficient antibody binding, only 29 muscle biopsies could be evaluated for Ki67 expression and 30 biopsies for NCAM expression. The cohort was divided by gender (20 women, 11 men). The groups differed significantly in maximal handgrip strength (*p* = 0.030) and the severity of sarcopenia represented by z-score sarcopenia (*p* = 0.040), showing that men were more affected by sarcopenia than women. Furthermore, men had significant lower MUNIX (*p* = 0.032) and higher MUSIX values (*p* = 0.030) than women in this cohort. Serum hormone concentrations for IGFBP-3 (*p* = 0.033) and ALS (*p* = 0.016) in male participants were significantly reduced compared to female participants. IGF-I levels did not differ significantly between gender (*p* = 0.296). No significant difference in the expression pattern of the histological proliferation marker Ki67 and denervation marker NCAM between male and female participants were found.

### Sarcopenia severity correlates with IGF-I concentrations

Figure 1 shows the relation between the degree of sarcopenia (represented by the z-score sarcopenia) and IGF-I serum concentrations. Pearson’s correlation coefficient was − 0.360 (*p* = 0.047), representing that patients with reduced serum concentrations of IGF-I are significantly more affected by sarcopenia. This effect does not remain after adjustment for sex (ß = − 0.299; *p* = 0.089).

### MUNIX and MUSIX measurements

Two participants did not tolerate the MUNIX procedure. Thirteen participants presented with pathological MUNIX values below the threshold of 80 (6 women, 7 men) and 7 participants (3 women, 4 men) with pathological MUSIX values above the threshold of 100 μV. Two MUSIX results had to be excluded from analysis due to unreliable measurement.

Figure 2 illustrates the relation between IGF-I concentrations and MUNIX (Fig. 2a) and MUSIX values (Fig. 2b). Pearson’s correlation coefficient between IGF-I concentrations and MUNIX values was ß1 = 0.512 (*p* = 0.005) and ß1 = -0.551 (*p* = 0.003) between IGF-I and MUSIX. These significant correlations remain after adjustment for sex (MUNIX: ß2 = 0.436, *p* = 0.014; MUSIX: ß2 = -0.545, *p* = 0.008).

### Ki67 and NCAM expression in dependence of IGF-I

Due to insufficient antibody binding, only the material of 29 muscle biopsies could be evaluated for Ki67 expression and material of 30 biopsies for NCAM expression.

Figure 3 shows the relation between IGF-I concentrations and Ki67+ mycocytes derived from muscle biopsy tissue (Fig. 3a), the histological marker for proliferation, and NCAM (Fig. 3b), a molecule that marks denervation/reinnervation processes in muscle tissue. Between IGF-I concentrations and the expression of Ki67 in myocytes a linear correlation was observed (ß1 = 0.424; *p* = 0.022), as well as between IGF-I concentrations and the percentage of NCAM expressing muscle fibers (ß1 = 0.380; *p* = 0.039). These linear correlations remained after adjustment for sex (Ki67: ß2 = 0.422, *p* = 0.028; NCAM: ß2 = 0.387, *p* = 0.043).

## Discussion

The main finding in our study is the significant association between IGF-I serum concentration, low numbers of motor units, NCAM and Ki67 expression. This finding emphasizes the important role of IGF-I on cell proliferation and reinnervation processes in aged muscle.

Our first finding in this study is the association of the severity of sarcopenia (z-score sarcopenia) in geriatric patients with reduced serum concentrations of IGF-I (Fig. 1). Since the pathogenesis of sarcopenia is believed to be multifactorial, endocrinological changes in aging play a key role in the multifactorial pathogenesis of sarcopenia. Research in mice and humans over the past decade has clarified the anabolic role of IGF-I as a growth factor in muscle metabolism [34] by promoting myoblast proliferation and differentiation and stimulation of satellite cell proliferation and muscle protein synthesis [35]. It had been concluded that reduced IGF-I concentrations in aging contribute to sarcopenia, i.e. the InCHIANTI study on 730 subjects showed that lower IGF-I concentrations were associated with the higher risk to develop sarcopenia [36]. We also demonstrate that correlation (Fig. 1) in our small sample of geriatric hip fracture patients, that shows a significant reduced IGF-I hormonal axis in total in our cohort independent from sex (Table 1) thus providing additional evidence for the assumption of an association between low levels of IGF-I and sarcopenia.

The second main finding in our cohort is the association of a reduced number of motor units in geriatric patients with reduced serum concentrations of IGF-I (Fig. 2a). Approaches to explain the age-related loss of muscle mass include the assumptions that either loss of muscle fibers due to denervation, degeneration of the neuromuscular junction, loss of motor units itself or all of those together are causative factors in sarcopenia [37]. Previous research on neuronal changes in aging muscle revealed a loss of motor units with age [38], whereas the remaining motor units increase in size as a compensation mechanism [39]. Drey et al. were the first to demonstrate motor unit loss in patients with sarcopenia [8]. They also used the MUNIX technique by Nandedkar et al. [29] to quantify the number of motor units in community-dwelling older adults. This electromyographic method using the hypothenar muscle as an ‘indicator muscle’ was initially developed for evaluation of disease progression in patients with amyotrophic lateral sclerosis [29], in which a progressive loss of first and second motor neurons occurs. Although we had examined older adults with acute hip fracture, the MUNIX technique is a valid method and previously has been shown to be applicable in patients with sarcopenia [12]. In our cohort, half of the included patients (51.6%) presented with low MUNIX values, meaning values below the threshold of 80, a cut-off suggested by Nandedkar et al. [30]. Our results show a significant association between low MUNIX values and a higher degree of sarcopenia, represented by a z-score sarcopenia (ß = − 0.411, *p* = 0.027, data not shown). We used this z-transformation including handgrip strength and muscle mass values with cut-offs according to the EWGSOP II criteria [27] to display the degree of sarcopenia in each patient and to classify the sarcopenia status even if patients were not entirely fulfilling the sarcopenia criteria. Our results support the previous findings of motor unit loss occurring in a geriatric population and are thus a conceivable contributing factor in sarcopenia development. The participants with reduced MUNIX values also show significant higher MUSIX values, representing the size of motor units. Since these participants were more affected by sarcopenia, this result supports our hypothesis of compensatory mechanisms in motoneurons such as neuronal sprouting. Since Drey et al. showed that the odds ratio for being sarcopenic with pathological MUNIX and MUSIX values is three times higher compared to a healthy control group [8], our results provide further evidence for MUNIX as a potential biomarker for sarcopenia caused by motoneuron loss and for its prospective clinical use in geriatrics. Nevertheless, the correlations could result either due to the relatively small sample size (*n* = 31) or it could be attributed to the remaining muscle fibers and their remaining motoneurons increasing in size [40].

Our data further demonstrate a possible link between endocrinological and neuronal changes in sarcopenia, as we found a significant reduced IGF-I hormonal axis in total in our cohort independent from sex (Table 1) as well as significant correlations of reduced IGF-I concentrations and low MUNIX values and MUSIX values in general (Fig. 2a and b). The neurotrophic role of IGF-I in the nervous system has been described before [41]. In particular, the hormone stimulates axonal sprouting and damage repair of motoneurons [22], thus contributing to reinnervation and regenerative processes, a decreased repair mechanism in age. Musaro et al. designed a transgenic mouse model expressing a full-length precursor of a localized IGF-I isoform, mIGF-I, which expression is normally induced after muscle damage. This muscle specific isoform maintains muscle hypertrophy and regenerative potential in aged muscle [18], supports recruitment of satellite cells in damaged muscle [42] and improves muscle mass and strength all by mediating associated pathways in muscle growth [34]. IGF-I supplementation therefore might be considered as a therapeutic drug in neuronal muscle wasting and sarcopenia. Neuronal sprouting after muscle fiber denervation seems to be limited in aging muscle tissue. This could be due to a loss of regeneration capacity or due to loss of possibility to signal the denervation status to surrounding muscle fibers. IGF-I might be one of the local stimulating hormone to support muscle and motor unit growth after denervation, but it is not yet known whether IGF-I also supports signaling. The significant correlation of higher MUSIX values with lower IGF-I concentrations (Fig. 2b) suggests that IGF-I either might not be involved in that local signaling or its concentration is too low.

To study regenerative potential of aged muscle, we measured the expression of Ki67 and NCAM in the muscle biopsy specimens. Ki67 protein is a widely used marker for cell proliferation in human tumor cells, especially in breast cancer. It is also commonly used as a marker in immunofluorescence staining techniques in detecting proliferating cells in any tissue including muscle cells [17, 43]. The neural cell adhesion molecule (NCAM) is a molecule expressed by muscle cells involved in muscle-neuron and muscle-muscle cell interactions. Its expression is regulated by the state of innervation of a muscle cell [44]. Accumulation of NCAM in a denervated and paralyzed skeletal muscle was first shown by Covault et al. [45]. Levels of NCAM expression since then display denervated muscle fibers in neurogenic disorders and aged muscle, even though Hendrickse et al. recently pointed out that NCAM may not be as useful as expected to detect denervation. His group studied the expression of NCAM upon time course of denervation duration and could not find a difference in NCAM levels in long term denervation compared to the control group after nerve injury [46]. However, NCAM stays a marker for early muscle fiber denervation. Our analysis in the muscle biopsy specimens reveals a significant correlation of reduced IGF-I concentrations in older adults and low Ki67 expression, thus a reduced amount of muscle fibers in a proliferating state (Fig. 3a). We further show, that if denervation occurs in aged muscle, the level of reinnervation (represented by NCAM expression in muscle tissue) seems to be correlating with IGF-I concentrations (Fig. 3b), indicating a connection of endocrine and neurological factors in the pathogenesis of sarcopenia.

It has yet not been described if IGF-I itself is able to directly upregulate the Ki67 protein on a molecular level, but it is known that IGF-I activates satellite cell growth and proliferation, therefore promoting muscle growth via the stimulation of the Akt/mTOR pathway [47]. Our findings support this anabolic role and justify the use of Ki67 as proliferation marker in aged muscle tissue. Further, our results demonstrate evidence for the hypothesis of failed denervation and reinnervation processes in sarcopenia, because the latter being dependent on sufficient IGF-I concentrations. The process of neuronal sprouting of mature axons as a rescue mechanism after nerve injury or denervation takes place in the entire nervous system and during all periods of life [48]. Kanda et al. reported restorage of the motor function in the medial gastrocnemius muscle of aged rates after nerve crush injury, suggesting that aged motoneurons preserve their capacity for axonal reinnervation [49]. In aging, motor unit loss occurs and results in denervated muscle fibers, which compensate the loss with reinnervation by axonal sprouting, a process called motor unit remodeling [50]. In turn, this leads to an increase in the size of motor units of the remaining motor units, as shown by the increased MUSIX values in our cohort. We found an association of high NCAM expression with higher IGF-I concentrations, stating that IGF-I might support the expression of NCAM. Gillon et al. [51] recently concluded that NCAM might be the decisive signal molecule for aged denervated muscle fibers to signal their denervation status to surrounding motoneurons. The ability to do so seems to be reduced in aged muscle, therefore missing the ability to reattract a neuronal input for reinnervation. In our cohort the NCAM levels significantly correlated to the IGF-I serum concentrations, showing a possible supportive role of IGF-I in reinnervation processes by supporting the potential to express in NCAM in aged muscle fibers after denervation in order to get reinnervated and prevent disuse atrophy. As we could only find NACM expression in muscle tissue in 12 of 31 muscle biopsies, this could be either due to the fact that these geriatric patients were just too ill to signal their denervation status and therefore lost the potential for reinnervation processes, or this failed reinnervation is due to a lack of systemic IGF-I. Nevertheless, this does not fully explain inverse correlation of MUSIX values, that represent the size of motor units, with IGF-I concentrations. Either denervation was not the main atrophy trigger in those patients or reinnervation failed in half of the cohort due insufficient IGF-I concentrations. Since an neuroprotective influence of IGF-I has been described [41], systemic administration of IGF-I has already been used in therapeutic trials in neurodegenerative and muscle atrophy associated diseases such as amyotrophic lateral sclerosis patients [24–26] and recently in older adults [52, 53]. Unfortunately, all studies in amyotrophic lateral sclerosis patients did not show any significant improvement in disease progression or muscle strength. One critical problem is the effective delivery of the neurotrophins to their target cells [54]. In turn, Butterfield et al. demonstrated increased muscle protein synthesis in response to one-month IGF-I supplementation in 14 older female adults [52], whereas Friedlander et al. could not find any changes in body composition, bone mineral density or handgrip strength after one-year IGF-I replacement [53]. These previous trials and results, combined with our findings, lead us to the assumption that sufficient IGF-I concentrations play a crucial role in muscle loss on the molecular level, in both endocrinological and neurological sarcopenia by establishing an anabolic environment. IGF-I supplementation should still be considered as a pharmacological treatment option in a subgroup of neurogenic affected patients with sarcopenia as it might promote the supportive endocrinological environment needed for sufficient muscle regeneration after denervation. Further studies on the regenerative capacity of aged muscle associated to IGF-I in sarcopenia would help to elucidate this potential anabolic treatment.

The main strength of our study certainly is the use of valuable muscle biopsy material of a geriatric patient cohort and the corresponding histological and electrophysiological examinations, including a comprehensive investigation of the GH/IGF-I axis.

However, some limitations should be noted. Firstly, the study is conducted in a cross-sectional study design which cannot prove causality. The correlations shown in the figures are just weak to moderate which is quite common in clinical samples. Secondly, we cannot entirely rule out misevaluation of sarcopenia status, as sarcopenia assessment was conducted right after surgery. Thirdly, given the relatively small study population, we cannot give general statements for a general population. Strictly speaking our results are only valid for hip fracture patients with sarcopenia and are therefore not generalizable for the whole group of patients with sarcopenia.

## Conclusion

To our knowledge, this is the first study to demonstrate a correlation of reduced IGF-I serum concentrations and markers of denervation in sarcopenia. Considering the small study population and the cross-sectional study design, a longitudinal observation of participants would be helpful to further elucidate these associations. Performance could be increased by a structured, continuous, and longitudinal bio-banking of muscle biopsies, especially in an orthogeriatrics setting. Our data open a new window for a discussion for IGF-I as a pharmacological agent in prevention and treatment in patients with sarcopenia, as it might be the vital hormone for establishing a sufficient environment for muscle regeneration in age by supporting neuronal reinnervation of denervated muscle fibers.

## Supplementary Information

**Additional file 1.**

## Data Availability

All data generated or analysed during this study are included in this published article.

## Abbreviations

aLM: Appendicular lean mass
ALS: Acid labile subunit
BIA: Bioelectrical impedance analysis
CMAP: Compound muscle action potential
EGWSOP: European Working Group on Sarcopenia in Older People
EMG: Electromyography
GH: Growth Hormone
ICMUC: Ideal case motor unit count
IGF-I: Insulin-like growth factor 1
IGFBP-3: Insulin-like growth factor binding protein 3
MUNIX: Motor unit number index
MUSIX: Motor unit size index
NCAM: Neural cell adhesion molecule
SD: Standard deviation
SIP: Surface electromyographic interference pattern
SMI: Skeletal muscle mass index
TBS: Tris-buffered saline
