# Supplementary material for: Influence of IGF-I serum concentration on muscular regeneration capacity in patients with sarcopenia
Source: BMC Musculoskelet Disord. 2021 Sep 20;22:807. doi: 10.1186/s12891-021-04699-3 (PMC8454138; doi:10.1186/s12891-021-04699-3)
Supplement: Supplementary file 1 — Additional file 1. [file 12891_2021_4699_MOESM1_ESM.docx]

**Appendix: Model for MUNIX**

Consider an ‘‘ideal’’ muscle with the following three characteristics.

(1) All motor units (MUs) are identical. This means that each Single Motor Unit Potential (SMUP) is identical in amplitude and waveform. (2) The Compound Muscle Action Potential (CMAP) is the sum of all SMUPs. Assuming there is no temporal dispersion, the CMAP waveform is a magnified image of all SMUPs. (3) Consider a Surface Interference Pattern (SIP) of a voluntary muscle contraction when a few MUs are activated. Assume that their SMUPs do not superimpose.

For the digitalised signal, $u_{i}$ is defined as the amplitude of SMUP at a certain time $t_{i}$. $N$is the number of motor units contained in the considered muscle. Index $j$in $u_{ji}$ identifies any SMUP, ranging from 1 to $N$. Therefore the first assumption can be translated into: $u_{1i}=u_{2i}=u_{i}$. The area of SMUP is computed by adding together the absolute values of $u_{i}$ multiplied by sampling interval $\Delta t$:

$Area(SMUP)=\sum_{i=0}^{t} \left|u_{i}\right|\bullet\Delta t$.

The power of a signal is computed by squaring its amplitude. Therefore the power of SMUP is calculated by:

$Power(SMUP)=\sum_{i=0}^{t} u_{i}^{2}\bullet\Delta t$.

Assume that $c_{i}$ is the amplitude of CMAP at a certain time $t_{i}$. Taking assumption 2 into account, $c_{i}=N\bullet u_{i}$. To calculate the area of CMAP, the same applies as with the area of SMUP:

$Area(CMAP)=\sum_{i=0}^{t} \left|c_{i}\right|\bullet\Delta t=\sum_{i=0}^{t} N\bullet\left|u_{i}\right|\bullet\Delta t=N\bullet\sum_{i=0}^{t} \left|u_{i}\right|\bullet\Delta t=N\bullet Area(SMUP)$. (a)

To calculate the power of CMAP, the same applies as with the power of SMUP:

$Power(CMAP)=\sum_{i=0}^{t} c_{i}^{2}\bullet\Delta t=\sum_{i=0}^{t} (N\bullet u_{i})^{2}\bullet\Delta t=N^{2}\bullet\sum_{i=0}^{t} u_{i}^{2}\bullet\Delta t=N^{2}\bullet Power(SMUP)$. (b)

Consider the area and power of SIP when a few MUs and their corresponding SMUPs $j=D$ are activated (assumption 3):

$Area(SIP)=D\bullet Area(SMUP)$, (c)

$Power(SIP)=D\bullet Power(SMUP)$. (d)

After some algebraic transformation of (a) – (d), it can be verified that:

$N=\frac{Area(SIP)}{Power(SIP)}\bullet\frac{Power(CMAP)}{Area(CMAP)}=ICMUC$. (e)

The aforementioned equation (e) reflects $N$ under the condition of the “ideal” muscle. This is indicated by the expression ICMUC (ideal case motor unit count). Nevertheless, the numerical value of ICMUC is quite reasonable given a low force and low SIP area, respectively (see assumption 3). In the model, MUNIX is defined as an ICMUC for an arbitrarily chosen low SIP area (20 mVms), where the assumptions are almost fulfilled. The relation of ICMUC to the SIP area is modelled by a power function:

$ICMUC=A\bullet(Area(SIP))^{\alpha}$.

The values of $A$ and $\alpha$ are derived from a regression analysis fitting a power function. Finally, MUNIX is calculated by:

$$MUNIX=A\bullet(20mVms)^{\alpha}$$
